# Food matrix and processing affect almond protein release during simulated digestion

**DOI:** 10.1186/2045-7022-1-S1-P20

**Published:** 2011-08-12

**Authors:** Giuseppina Mandalari, Giuseppe Bisignano, Martin Wickham

**Affiliations:** 1 Institute of Food Research, Norwich, UK; 2 Uiversity of Messina, Messina, Italy; 3 Leatherhead Food Research, Leatherhead, UK

## 

Understanding the fate of proteins during digestion is of especial relevance to understanding the basis of food allergies. Little is known of the immunological mechanisms involved in the sensitisation of an individual towards a food and, with the exception of the fruit and vegetable allergies (which appear to be secondary responses to tree and weed pollen allergies), it is thought that food allergens (or fragments thereof) must cross the gastrointestinal (GI) mucosa in order to interact with the immune system. This is also a prerequisite for an allergen to elicit a reaction in an individual who has already become sensitised. When food is ingested it is crushed and sheared in the mouth where it is mixed with saliva, subjected to gastric processing for a variable period where the pH may fall to as low as 2 and on entering the small intestine it is neutralised and subjected to the duodenal, jejunal and ileal environments on its passage to the large intestine. During all these phases it is mixed with enzymes (amylases, proteases, lipases) and in the duodenum to detergents (bile salts).

Here we describe the release of almond protein during simulated GI digestion and the effects of food matrix and processing on its release. A Dynamic Gastric Model (DGM) was used to represent the in vivo physiological conditions of the gastric environment with addition of acid secretions, gastric enzymes and surfactants. Results obtained by SDS-PAGE analysis and HPLC showed a slower kinetic of protein digestion when almond flour was incorporated within a chocolate dessert and a Victorian sponge. In-gel tryptic digestion coupled with MALDI-ToF/ToF mass spectrometer was used to follow the rate of almond protein digestion in the food matrix.

Food matrix and processing affect digestibility of almond protein in the upper GI tract.

This work has been funded by the Almond Board of California.

